# Political legitimacy and European monetary union: contracts, constitutionalism and the normative logic of two-level games

**DOI:** 10.1080/13501763.2014.995118

**Published:** 2015-01-22

**Authors:** Richard Bellamy, Albert Weale

**Keywords:** Democracy, euro crisis, legitimacy, political constitutionalism, social contract theory

## Abstract

The crisis of the euro area has severely tested the political authority of the European Union (EU). The crisis raises questions of normative legitimacy both because the EU is a normative order and because the construction of economic and monetary union (EMU) rested upon a theory that stressed the normative value of the depoliticization of money. However, this theory neglected the normative logic of the two-level game implicit in EMU. It also neglected the need for an impartial and publically acceptable constitutional order to acknowledge reasonable disagreements. By contrast, we contend that any reconstruction of the EU's economic constitution has to pay attention to reconciling a European monetary order with the legitimacy of member state governance. The EU requires a two-level contract to meet this standard. Member states must treat each other as equals and be representative of and accountable to their citizens on an equitable basis. These criteria entail that the EU's political legitimacy requires a form of *demoi*cracy that we call ‘republican intergovernmentalism’. Only rules that could be acceptable as the product of a political constitution among the peoples of Europe can ultimately meet the required standards of political legitimacy. Such a political constitution could be brought about through empowering national parliaments in EU decision-making.

## THE MAKING OF THE LEGITIMACY CRISIS

The crisis of the euro area (EA) has severely tested the political authority of the EU. Since 2010 the EU and its members states have been forced to improvise policies and processes to deal with the crisis, including the European Semester, a strengthened Stability and Growth Pact, the Treaty on Stability, Co-ordination and Governance, the European Financial Stability Facility (EFSF) and its successor in the European Stability Mechanism (ESM) (Begg 2013; Ioannou *et al.* 2015). The European Central Bank (ECB) has embarked upon two rounds of long-term refinancing operations to improve bank liquidity, in effect buying sovereign debt, as well as announcing its willingness to engage in outright monetary transactions (OMT), a policy allegedly leading Jens Weidmann, President of the Bundesbank, to say that this is tantamount ‘to financing governments by printing banknotes’ (Steen 2012). And still the prospect of deflation looms over European economies (House of Lords 2014c: 13 and *passim*).

The same conditions that gave rise to these policy imperatives have required the EU to find ways of supporting the governments of Greece, Ireland, Portugal, Spain and Cyprus in defiance of the no bail-out clause of the original monetary union (now Article 125 of the TFEU). They have resulted in the Troika imposing restrictions on the national budgets of debtor governments, policies that have been resisted by national parliaments and opposition movements. They have strengthened anti-EU parties, with a record number of Eurosceptic Members of the European Parliament (MEPs) elected in the European elections of May 2014. They have provoked legal actions in national constitutional courts in both creditor countries like Germany (Federal Constitutional Court 2014a, 2014b) and debtor countries like Portugal^1^ resulting in judgements that question the legitimacy of the programmes. They have stimulated continued, and sometimes violent, demonstrations against public expenditure austerity packages. They have entailed the installation of technocratic governments in Greece and Italy in 2012 as a way of dealing with the inadequacies of their respective political institutions, as well as the electoral defeat of incumbent governments in Spain and France. In short, they have brought about a crisis of political legitimacy for the EU.

The Lisbon Treaty was widely regarded as having settled the institutional architecture of the EU after nearly two decades of constitutional debate. The EA crisis has reignited those issues. The new policies and processes that have been inaugurated have changed the balance of power within the EU and opened up questions about what ‘deep and genuine’ economic and monetary union (EMU) requires by way of institutional change (European Commission 2012; House of Lords 2014a). In these debates, issues of normative political legitimacy inevitably arise, because the EU is a normative order. That is to say, the agreements that it embodies contain principles and values defining norms of behaviour for member states and EU institutions. The Treaty on European Union (TEU) and the Stability and Growth Pact (SGP), strengthened through Title VIII of the Treaty on the Functioning of the European Union (TFEU), together with the Six-Pack and the Two-Pack, have required member states to make progressively stronger commitments to one another in respect of economic and fiscal policy (Ioannou *et al*. 2015). Those commitments have been reinforced by the Fiscal Compact contained in the Treaty on Stability, Co-ordination and Governance in the Economic and Monetary Union (TSCG), by which member states have undertaken to ensure that national budgets are in balance or in surplus ‘through provisions of binding force and permanent character, preferably constitutional, or otherwise guaranteed to be fully respected and adhered to throughout the national budgetary processes’ (TSCG, Article 3.2). Such measures provide a set of rules and principles by reference to which policies and institutional change are justified. Resting on agreed norms and principles, they form a political contract among member states.

However, questions of normative legitimacy are raised by the crisis not simply as a result of the EU's being a normative order, but also because the construction of EMU rested upon a set of constitutional principles that contained strong – and contestable – normative assumptions. In particular, economic and monetary union was constructed according to the principles of legal constitutionalism (Issing 2008; James 2012). Legal constitutionalism is a political doctrine to the effect that a legitimate political regime must rest on a set of legal rules that constrain the actions of politically responsive decision-makers. In some versions (e.g., Dworkin [1996]) such restrictions take a ‘left liberal’ form; in others (e.g., Hayek [1979]), they take a neoliberal form (see Bellamy [2007]). Our contention in this contribution is that the developing political contract underlying EMU has produced restrictions on member states with respect to their public budgets that amount to more than simply a treaty agreement; they have given rise to a treaty agreement underwritten by the principles of legal constitutionalism of a neoliberal kind, indeed of a specific kind within neo-liberalism.

The tradition of political analysis that fed into the construction of the single currency and its management is to be found in the work of thinkers associated with the Hayekian version of constitutional liberalism (see James [2012: 6–7]). According to this tradition, democratic governments have a tendency to fiscal irresponsibility owing to politicians having incentives to buy votes through excessive public expenditure. In seeking re-election, political representatives are motivated to respond to the wishes of special interest groups in the short term rather than framing legislation for the public interest in the long term. Particular manifestations of these tendencies might include the provision of price support schemes for agriculture, the protection of domestic industry from foreign competition, interference in controlling the terms of employment contracts that can be agreed, and expenditure on public works that benefit only localized constituencies. Hayek (1979) held that, to avoid these pitfalls, states need to be constrained by constitutional rules and mechanisms from engaging in excessive expenditure and unduly interfering in the operations of the free market. Behind the construction of the specific set of rules for EMU, therefore, lay a more general set of premises concerning the character of a democratic political order.

The problem with this construction, we argue, is that, when applied to EMU, it neglects the normative logic of two-level games. According to this logic, when governments make commitments to one another about their future behaviour, they *simultaneously* need to be responsible and accountable to their domestic populations in order to retain their political legitimacy. The logic of two-level games was originally developed by Putnam (1988) to account for the outcome of the Bonn economic summit of 1978, and has been subsequently applied to empirical cases ranging from security issues to economic diplomacy and North–South relations (Evans *et al.* 1993). As Pollack (2001: 225) has pointed out, it also lies behind liberal intergovernmentalist accounts of EU integration such as that of Moravcsik (1998) and Schimmelfennig (2015). However, this framework of analysis neither implies fixed preferences (Crespy and Schmidt 2014), nor does it have only an empirical use. Beyond its empirical applications, the logic of two-level games also has a normative interpretation (Savage and Weale 2009) providing a model by which we can evaluate the justifiability of constitutional arrangements.

The neglect of the normative logic of two-level games in the construction of EMU is compounded by a second problem within legal constitutionalism: namely, its disregard of the existence of reasonable differences in political judgement over the principles that should govern a monetary union made up of different sovereign states, each with their own traditions of economic and monetary policy. Indeed, even within the broadly neoliberal tradition of thinking about economic constitutions, there are important differences of substance as well as emphasis. When the conditions for continuing contestation over policy measures and organization exists, the putative political legitimacy of EU legal constitutionalist arrangements, such as those underlying EMU, the SGP and the TSCG, reinforce the practical contradiction of the two-level game implicit in the economic constitution. By contrast with this attempt to entrench legal constitutionalism, we suggest that the design of an economic constitution ought to respect the principles of political constitutionalism, with its requirement that governments be responsive to the public reasoning of their citizens within the continuing democratic conversation that makes up a political society (Bellamy 2007).

In pursuing this argument, the contribution proceeds as follows. In the next section we lay out the normative logic of the two-level game embodied in the construction of EMU. According to this logic, those participating in international agreements have a dual duty: to deal fairly with one another, on the one hand; and to be responsive and accountable to the democratic reasoning of the people whom they represent, on the other. In acknowledging this dual duty, they should also acknowledge that their fellow negotiators have a similar duty in respect of their own peoples. The penultimate section indicates why, given reasonable disagreement about the principles that should govern an economic constitution, the legitimacy of EMU cannot be simply secured by framing the related fiscal rules in legal constitutionalist terms. The long-term legitimacy of EMU is compatible only with political constitutionalism. We conclude that so long as the EU remains subject to the logic of delegation implicit in the normative logic of two-level games, EMU must remain subject to the equal control and influence of the different member state *demoi* – a position we characterize as ‘republican intergovernmentalism’. We suggest this result can be achieved through the empowerment of national parliaments in EU policy-making.

## THE NORMATIVE LEGITIMACY OF TWO-LEVEL CONTRACTS

At the centre of the issue of political legitimacy is the question of the credibility, and consequently the justifiability, of the reasoning underlying the norms and principles on which the construction of EMU is based. Yet, how might one evaluate such credibility? We approach this question through contractarian political theory. According to contractarian theory, political authority is to be understood as arising from a contract to mutual advantage implicitly or explicitly agreed among the members of a political association. The need for political organization can be modelled as the solution to dilemmas of collective action (Buchanan and Tullock 1962; Gauthier 1986; Ostrom 1990; Weale 2013). These dilemmas occur when unco-ordinated action by separate agents gives rise to potential gains from co-operation, as in an agreement on weights and measures or the rules of the road, or where unco-ordinated individual action leads to harmful side-effects from otherwise legitimate human activity, of which pollution and resource depletion are the obvious examples. If we think of political associations as having a contractarian logic in this sense, then we can address the issue of credibility by asking what conditions have to be satisfied for actors to find a contract that they can rationally support (Gauthier 1986).

The general logic of contractarian analysis can be applied not only to the study of natural persons but also to relations between states. States can impose harmful externalities on other states and their populations through cross-boundary pollution, trade restrictions or population movements. They can also fail to secure common advantages through a lack of political co-ordination. The EU has often been portrayed as a mechanism for overcoming these problems in the international arena (Moravcsik 1993). The assumption is that the policies that fall within the competence of the EU are in the long-term common interest of the member states, offering Pareto improvements over a prevailing *status quo* for all concerned. However, many such issues are subject to the logic of the prisoner's dilemma. Each member state may be better off with an agreed policy with which all other member states comply but with which it does not, than it would be when it complied as well, even if all would be worse off without any agreement. Yet, if this logic is clear to all, none would rationally comply and so the policy will either never be agreed or will unravel over time. Thus, the fundamental problem to be solved in any political contract between states is that of inducing credibility in others of one's commitment to the policy to be agreed to avoid defection from a mutually beneficial agreement. To overcome this free rider problem requires states to be able to make credible commitments to one another about their willingness to fulfil their obligations, even on those occasions when fulfilling those obligations proves onerous.

The logic of the N-person prisoner's dilemma was reflected in the construction of EMU. As Issing (2008: 234–6) has clearly explained, it was thought that, because democratic competition works to create deficit financing, thereby undermining the long-term stability of the currency and public finances, the euro was designed to represent depoliticized and hence stable money. On this analysis, the political benefits of deficit spending in the form of votes gained by governing parties are enjoyed by national players, while the potential negative effects, notably higher interest rates, are felt by all states. So, it is rational for prudent states to seek to ensure that they do not incur the spillover effects of others’ deficit spending, and they can attempt to do this by institutionalizing a no bail-out rule. The alternative to such a rule is to leave discipline to the markets. However, within a currency union there is no exchange rate risk to a national government from deficit financing, and so borrowing premiums remain low over a period of time and credit risk builds up (Issing 2008: 193–4). Aware of this possibility, no rational state would prudently enter into a currency union without a no bail-out rule. Hence, in order for any such agreement to take place, states must commit to funding their own borrowing. Each state has to be able to make a credible commitment to other states about the maximum deficits that they are willing to tolerate in their public spending plans. This, in short, was the rationale of the no bail-out clause of the Maastricht Treaty. The SGP arose from the recognition that the Maastricht rules of no bail-out and no exit were insufficient to prevent member states continuing to run excessive deficits. The idea was that the scope for fiscal adjustments among participating states had to be defined once and for all. Political representatives at the member state level could still co-ordinate fiscal and monetary policy, but only on condition that the monetary component was fixed exogenously by an independent European Central Bank, the ECB, that had been deliberately isolated from political interference (see Issing [2008: 193–5]).

When Germany in 2002 and then France and Germany in 2003 breached the provisions of the SGP, member states within the contract of monetary union had an incentive to strengthen monitoring and compliance even more. With the coming of the financial crisis, the next stage of the contractarian logic was to embed the SGP in the European Semester, together with the Six-Pack and the Two-Pack, the effects of which were not only to increase the intensity of the monitoring of budgetary plans, but also to ensure co-ordination among member states *before* those plans were put to national parliaments. The Fiscal Compact, the aim of which is to alter the institutional structure of domestic political arrangements to prevent excessive deficits from arising or rectify them as quickly as possible if they do exist, reinforces these provisions. As contractarian theory predicts, these devices emerge where previous commitment has been shown wanting and there is no alternative to continuing collective association. In other words, when commitments turned out not to be credible, the contractarian logic leads actors to a search for greater compliance by increased monitoring, penalties and institutional restructuring (Weale forthcoming).

Does this contractarian rationale provide a justification of the political legitimacy of EMU as it has been constructed? It could only do so provided that the states in question could be regarded as unitary actors. Yet, treating states as unitary actors is merely a simplifying assumption, useful for the purposes of some types of analysis but distorting if taken as an accurate representation of an empirical situation. States are collective entities made up of constellations of many actors. In political associations modelled according to the norms of two-level games, the political representatives of each state simultaneously owe obligations to the political representatives of other states and to their own populations (Savage and Weale 2009), with implications for their ability to comply with their contractual commitments.

The credible commitment that each state has to be able to make to every other concerns such matters as the maximum budget deficits that they will allow in their public spending plans, the rate at which deficits will be rectified and the balance between the growth of GDP and the growth of public expenditure. However, the commitment of states with regard to these policy strategies can only be made credible provided that each state enjoys the confidence of its citizens. Only with the confidence of their citizens will these states possess the capacity to implement the policies implied by the international agreement. In the modern world, this confidence and the resulting capacity to implement policy rest upon democratic political legitimation. Monetary union implies, then, that each state can have the confidence that all other states can secure sufficient ongoing domestic support to meet their consequent obligations. Hence, only if states enjoy democratic legitimacy will other states have reason to believe that their commitments are credible.

A similar interlocking logic arises in the relationship of states to their citizens. For international agreements to be credible, the governments responsible for implementing them must be able to give domestic populations good reasons for compliance, showing how an agreement will serve the collective interest. At the same time, each state must recognize that all other states that are parties to the agreement are similarly acting as representatives of their citizens. The state parties are thus engaged in a two-level game, in which the terms of the agreement have to be simultaneously acceptable to other negotiating parties *and* to their domestic constituents. Simultaneity in this context does not mean 'occurring at the same time', but indicates that any international agreement must fulfil two sets of conditions. First, an international agreement requires 'fair dealing' among states in their relations with one another as the representatives of their peoples. Second, states must ensure the general acceptability of the agreement to their respective peoples and be able to justify their international commitments, including any provisions for side payments, as being a reasonable way of advancing their joint and several common interests. Unless this second condition is met, so that a state can guarantee the backing of the people it represents, no other state party to the putative contract can be confident that a commitment made to it is credible.

In short, the logic of collective commitment in a monetary union presupposes the logic of political democracy at the national level. Unless all the state parties to an agreement possess a credible democracy at the national level, it is a practical contradiction at the international level for them to enter into commitments with each other, since, in those circumstances, no state could rationally trust the commitments of the other states or be trustworthy itself. Consequently, *pace* certain analysts of the EU (Majone 2001; Scharpf 1999) input legitimacy at the domestic level cannot be substituted by output legitimacy at the international level – particularly if the beneficial effects of those outputs vary over time and between the different parties to the agreement in ways that might be regarded as unfair (Bellamy [2010]; a point acknowledged by the post-crisis analyses of Majone [2012] and Scharpf [2011]). Therefore, the search for ‘an ever closer union of the peoples of Europe’ is in effect a search for credible commitment devices among the contracting member states in respect of the peoples whom they represent (Bellamy 2013).

The need for domestic political legitimacy is not simply a political fact; it is also a reason within a normative order. An international agreement involves each state recognizing that all other states are embedded within a normative order that governs their internal and external relations. Consequently, each state requires democratic legitimation for its commitments. The most elaborately worked out example of the logic of such a normative order is that provided by the German Federal Constitutional Court in its jurisprudence on EMU starting with *Brunner* (Federal Constitutional Court 1993). That jurisprudence recognizes that the German state needs to be able to enter into long-term international commitments in order to be able to secure benefits that are only available through internationally co-ordinated action. At the same time, the jurisprudence of the Court insists that any international commitment must be consistent with those principles of the Basic Law that bind the German state in perpetuity to the principle of democratic authority stemming from the people. In particular, the voting rights of German citizens should not be compromised by the German parliament losing meaningful control over the direction of economic policy. Therefore, the Court has seen its task as being to make it legally and constitutionally possible for the German state to enter into and honour international agreements that are in its interests and in the interests of other states who are party to the agreement, whilst at the same time retaining the principle of the democratic self-determination of the German people that is a fundamental element of the Basic Law. In a series of judgements, the Court has reasoned that these different demands can be reconciled through the doctrine of delegation. So long as the international agreement could be said to rest on the delegated authority of the member state and the *Bundestag* retained the power of revoking Germany's participation in the international agreement, then the principle of democratic self-determination was respected.

As Gustavsson (1998) noted, the Court's reasoning in *Brunner* rested upon three assumptions about EMU: its revocability by the *Bundestag*; its marginality in terms of the scope of obligations it implied; and its predictability. The subsequent jurisprudence of the Court has had to deal with the failure of one or more of these assumptions to obtain in practice. Thus, in a recent judgement on the constitutionality of the policy of OMT by the ECB (Federal Constitutional Court 2014a), a majority of the judges ruled that OMT were unconstitutional, because they involved an open-ended commitment by the German government. In other words, the scope of the obligations implied by OMT was neither limited nor predictable. Although the Court referred the matter to the Court of Justice of the European Union, it offered its own (sceptical) interpretation of the compatibility of the ECB's planned action with treaty and constitutional requirements. However, the kernel of its judgment turned on the force of Article 38 (1) of the German Basic Law. In line with its previous jurisprudence, the Court interpreted this Article as requiring that state authority could not be transferred to the extent that democratic control becomes nugatory. The right to vote is in effect defined as the right to vote in an election where the result will lead to meaningful parliamentary control over the conditions of collective life, thereby expressing the self-determination of the people. Democratic self-determination means that the scope of the *Bundestag's* authority cannot be rendered nugatory, and, if the German government failed to contest the policy of OMT, then its actions can be revoked (for this logic, see also Lindseth [2010: 24]).

On many matters of international agreement, domestic acceptability can be presumed by national decision-makers because the issues involved are technical, have low political salience or can be negotiated with the agreement of specific interest groups who share a consensus on which polices best serve their mutual advantage. In other words, they satisfy something like a marginality requirement. Prior to EMU, the EU's competences largely concerned such low salient issues and hence aroused comparatively little democratic contestation (Moravcsik 2002). However, the logic of monetary union does not fall into any of these categories. Although it is technical, its ramifications are wide. Few items are as politically salient as the reliability of a nation's currency. And interest groups typically take different and incompatible positions on the desirability of different monetary policies. In these circumstances, the assumption that states are acting as authorized representatives of their populations will break down, unless there are good reasons for thinking that the authorization is open-ended (hence the shift in the post-crisis analyses of Scharpf [2011] and Majone [2012], which, unlike Moravscik [2012], have moved close to the argument made here). However, as the jurisprudence of the German Federal Constitutional Court shows, after 1993 no other state had reason to think that the authorization was open-ended in the case of Germany. It was predictable that at some stage the limits of monetary integration would be met. This line of argument can be generalized. For just as other states had no reason for thinking that Germany would have an irrevocable commitment to all the implications of EMU, so no one in Germany could reasonably think that all other states could retain a democratic mandate for abiding by the rules of EMU when those terms became unpredictably onerous.

The practical contradiction at the heart of EMU is that member states could only find the terms of the contract credible on condition that they could assume that the commitments entered into by all other member states went beyond the scope of democratic legitimation within those states. That the contradiction revealed itself in the instability of the political contract on which EMU rested arose in part from the predictable unpredictability of monetary union. That feature in turn stemmed from the fallibility of political judgement within the circumstances of politics, an element of the normative logic that we discuss in the next section.

## LIBERALISM VERSUS LEGAL CONSTITUTIONALISM

Legal constitutionalism of the sort that underlies the constitution of EMU represents one tradition within the liberal inheritance, one that is notably counter-majoritarian in its implications. According to that tradition, if modern democracies have the characteristics attributed to them by neoliberal legal constitutionalists, these commitments could not be credible, since the governments of the same states that entered into the contract would be prone to myopic and short-term sectional pressures such that they would take any opportunities that might arise to free ride on the co-operation of others. If the temptation to free ride is built into democratic governments in this way, then there is no credible basis for commitment on the part of any potential party to the contract. The only basis for a credible agreement on monetary union would be through the general establishment of legal economic constitutions at the national level, underpinned by powerful counter-majoritarian institutions, so as to break the link between public expenditure and responsiveness to the preferences of the population. Of course, this proposal is an implication of the neoliberal legal constitutionalist analysis, and the first steps along such a path are embodied in the requirements of the TSCG.

However, counter-majoritarian legal constitutionalism in the economic realm is only one way of reading the liberal inheritance. Indeed, that tradition is at odds with another liberal idea: namely, the claim that any constitutional political contract should recognize the ‘burdens of judgement’ in its construction (Rawls 1996: 54–8). The burdens of judgement arise from such general features of human judgement as the complexity of empirical evidence, the different weight that different persons will put on different types of evidence, the vagueness of relevant concepts and the problems of assessing evidence. Given the burdens of judgement, a constitution should refrain from imposing requirements on those subject to it that will be matters of reasonable disagreement, matters, in other words, in which no knockdown arguments are possible. Rawls used this argument to exclude the constitutional entrenchment of religious doctrines because they rested on controversial philosophical premises, an issue that also arose in the convention on the putative EU constitution (Olsen 2004). However, Rawls (1996: 225) also gives the example of disputed ‘elaborate economic theories of general equilibrium’ as involving inherently controversial views that should not be given constitutional status. If one takes this view of disputed economic theories, the fair value of political liberties cannot be maintained if some views are given a privileged constitutional position *vis-à-vis* other views.

Does the entrenchment of a particular form of Hayekian theory in the constitution of EMU fall foul of this condition? There are a number of reasons to suppose that it does. Firstly, Hayek himself opposed EMU in part because he recognized economic policy, even of a libertarian kind, was not a matter that could be legally entrenched. Instead, he advocated free competition between rival currencies provided by private rather than public banks (Hayek 1978). Although this is a position that Issing (2000) attempted to contest on neo-Hayekian grounds, Hayek's scepticism about EMU was a logical consequence of his belief that viable economic orders were the evolutionary product of human action but not of human design (Hayek 1979). In other words, the attempt to construct an international monetary order by political fiat would replicate the fallacies of central planning on which the road to serfdom was based.

Secondly, even within neoliberalism, there are other traditions of theory that take a non-evolutionary view of the economic order. Although sometimes identified with a Hayekian perspective, even by Hayek (1967: 252–3) himself on some occasions, German ordoliberal economists like Eucken and Röpke, took the view that a functioning economy presupposes a moment of constitutional founding in which the rules of its operation are determined (Eucken [1951a, 1951b]; compare Goldschmidt [2000]; Nicholls [1994]; Peukert [2000]). As various commentators (for example, Sally [1998]; Streit and Wohlgemuth [2000]) have noted, this ordoliberal tradition contrasts with the Hayekian position in being rationalist and constructivist. It presupposes that the institutional form of the economy is determined within an already established legal order and political community. Economic integration is not an instrument to create a political community, but an expression of the political choices of that community.

Thirdly, this ordoliberal view is consistent with the worries many economists and policy-makers had expressed about the sequencing of European political union and monetary union and the design flaws built into EMU before the euro crisis had revealed these problems. For example, in a paper summarizing a wide range of work, Bordo and Jonung (2003: 43–4) pointed out that EMU lacked both a lender of last resort, by contrast with other modern monetary systems where central banks were able to ensure liquidity, and a central authority to supervize financial systems, including the commercial banks. They went on to point out that the absence of any central co-ordination of fiscal policies within EMU combined with ‘unduly strict criteria for debt and deficits … implies that EMU will not be able to respond to asymmetric shocks and disturbances in a satisfactory way’. Finally, and as many other economists also noted, they pointed out that Europe is too large and diverse an area to form a well-functioning currency union, with the efficiency gains from increased trade not large enough to outweigh the costs of surrendering control over national monetary policies.

Fourthly, it is well established that different national traditions of economic policy-making fed into the creation of EMU. For reasons of history and intellectual tradition, German policy-making gave pride of place to the goal of price stability underpinned by the independence of the central bank. By contrast, French thinking gave priority to *gouvernement économique*, a view of the relationship between government and the economy in which executive action played a large role in securing the day-to-day steering and co-ordination of the economy, as well as providing capital for investment in major projects (Dyson and Featherstone 1999; Jabko 2006: 168–72). Historically and institutionally rooted traditions do not disappear in a new policy framework, but manifest themselves in different ways. In particular, when it comes to questions of how countries recover from large economic shocks, there will be differences in what is seen as justifiable requirements; for example, how quickly and by what methods to re-establish internationally credible debt levels within the framework of the Excessive Deficit Procedure. Similar differences of judgement will affect how countries think about the institutionalization of debt brakes and other constitutional devices under the TSCG.

The implication of these points is that legal constitutionalism presupposes that there can be agreement on the basis of the constitutional essentials of a European monetary order, although the epistemic conditions do not exist to establish that agreement. Indeed, even the German *Bundesbank*, so often presented as a model apolitical central bank, had its independence from the German government tested both by Adenauer and Schmidt (Kennedy 1991: 37–42). If within a single country, with powerful political and intellectual traditions justifying a strong independent central bank, the issue can be contested, it is not surprising that a rigid pan-European economic constitution based on the idea of automatic rules will be contested even more.

## POLITICAL CONSTITUTIONALISM AND EUROPEAN ECONOMIC GOVERNANCE

The argument so far may be summarized as follows: credible commitment by governments at the international level presupposes political legitimacy at the domestic level; but the domestic legitimacy of democratic governments in turn presupposes that commitments may be modified or altered through political processes. Moreover, the epistemic conditions arising from the burdens of judgement reinforce the need for open discussion and democratic deliberation. Legal constitutionalism at the international level, therefore, risks undermining rather than reinforcing the credibility of state commitments if the measures legally entrenched are matters that should be subject to ongoing political debate by domestic electorates.

Political constitutionalism offers an alternative to legal constitutionalism (Bellamy 2007). By contrast to legal constitutionalism, political constitutionalism contends the terms of the political contract must be subject to ongoing debate among citizens with regard to both the procedures of decision-making and the substance of decisions. Judgments about either cannot be legitimately entrenched or handled by judicial or technical bodies that are isolated from democratic processes because such isolation fails to recognize the equal legal and political status of citizens. Political constitutionalists argue that the functional complexity, ethical diversity and openness of liberal societies make individual judgements about the public good inevitably partial and fallible. Because we are inescapably limited in our knowledge and experience, even the most conscientious persons will tend to reason from their own values and interests and be prone to error with regard to the present and future interests of others. If the collective decisions needed to regulate social life are to be not only impartial but also well informed with regard to the views and circumstances of those to whom they apply, so that they treat citizens with equal respect and concern, then citizens must have equal influence and control over the direction of public policy. *Pace* neoliberal thinkers, such as Hayek, such equal influence and control cannot be provided by markets but only by a democratic process, albeit indirectly through the election of decision-makers (Bellamy 1994).

Legal constitutionalism in its purest form tries to place the legal and political system itself and even many public policies beyond political contestation, defining in substantive and concrete terms how both might be best configured so as to realize equal concern and respect. By contrast, political constitutionalism in its purest form regards legitimacy as dependent upon the ability to employ existing political procedures to contest the procedural and substantive adequacy of the democratic system and its policies through the constant struggle of citizens to exercise equal influence and control over both. Most liberal democracies combine different degrees of each of them, some nearer to the political constitutionalist end of the spectrum and others more at the legal constitutionalist end. The various member states manifest considerable diversity in this respect, making all but the most abstract and procedural forms of legal constitutionalism difficult to agree. Hence the need for political constitutionalism between even those member states that have legal constitutionalist regimes (compare Glencross [2013]).

From the perspective of the normative logic of two-level games, the legitimacy of the integration process depends on its taking the form of what might be termed ‘republican inter-governmentalism’ (Bellamy 2013); that is, the governments and their agents can only enter into credible commitments with each other to the extent that they possess ongoing democratic authorization to represent their respective peoples, and acknowledge the equal right and obligation of all the other governments to represent their peoples (Pettit 2010). This logic stands behind the largely consensual character of much EU decision-making, not least the unanimity rule for any treaty change and the need for such changes to obtain domestic ratification within all 28 member states. Such features have led a number of commentators to remark on how the EU is best characterized not as a democracy, with EU citizens forming a pan-European demos, but as a *demoi*cracy between the different people*s* of the member states (Chevenal and Schimmelfennig 2013; Nicolaidis 2013).

We have argued that the legal constitutionalist mechanisms embodied in the TSCG cannot provide EMU with political legitimacy of a normative kind. It is not possible to model the choices of the actors according to the normative logic of the two-level contract in such a way that their practical reasoning is credible. If such reasoning cannot be modelled in a contractarian way in theory, it will not be credible in practice. Instead, EMU must remain part of the political constitution provided by the ongoing democratic influence and control of those subject to it. Within the EU as presently constituted, this political constitution must reflect the normative logic of two-level games. As such, political legitimacy comes not from a single EU *demos* but from an agreement among the different *demoi* of the eurozone, as negotiated by their elected representatives. For EMU to be legitimate, therefore, it must be under the *demoi*cratic control of European states. The logic here is that of the delegation of authority, with the problem of democratic legitimacy in the EU, not that of the democratic deficit but that of the democratic disconnect – the failure to ensure policy-making remains under the equal influence and control of the constituted peoples of the Union via their domestic democratic processes (Lindseth 2010: 234).

Can such *demoi*cratic control be achieved in the case of a currency union? A detailed response lies outside the scope of this contribution. Here, we wish merely to indicate the institutional structures needed to place EMU under a political rather than a legal constitution, and to note how these structures exist within the EU to a sufficient degree for this proposal to be plausible. The main lines of such an approach can be found in the German Constitutional Court's judgements from 1993 onwards referred to earlier. According to the Court, the national parliament, the *Bundestag*, as the representative body of the German people, plays an integral role in realizing the ‘right to democracy’ guaranteed by the German Constitution. Moreover, its budgetary responsibilities form an intrinsic aspect of that role, given that decisions on revenue and expenditure constrain the choice of public policies that shape the collective life of citizens. Adopting reasoning that encapsulates both political constitutionalism and the *demoi*cratic approach, the Court has argued that ‘sufficient space’ has to exist for the citizens of the member state to be able to interpret the fundamental rights that underlie their ‘economic, cultural and social living conditions’. Given reasonable disagreements about the relative importance and nature of these rights and how they might be best interpreted and realized – disagreements that have been resolved in different ways over time within each of the member states, as their different political and constitutional traditions attest – European unification could not be conducted in such a way as to leave no space for the *demoi* of the contracting parties to determine their collective life according to their differing ‘cultural, historical and linguistic perceptions’ through ‘public discourse in the party, political and parliamentary spheres of public politics’ (Federal Constitutional Court 2009). As a result, the Court has insisted on the *Bundestag's* right of participation in ESM, particularly in authorizing extensions of the guarantees for the fund (Federal Constitutional Court 2014b).

Drawing on this reasoning, two roles for national parliaments emerge within EMU. The first, domestic, role is to ensure that in negotiating budgetary rules at the EU level, the elected executives of each of the contracting member states act on the authority of their national parliament, and that the subsequent undertakings remain subject to their control and scrutiny. There are signs that other national parliaments are following the German lead. For example, Spain has set up a parliamentary budget office – the *Oficina Prespuestaria de las Cortes Generales* – that checks and assesses the execution of the budget and provides information to the legislature. The French and Italian Parliaments have likewise requested higher standards of information and transparency on issues of European economic governance. The second, inter-parliamentary, role involves national parliaments working together to ensure that EU measures treat each of the member states with equal concern and respect as self-governing polities. That role was developed formally with Lisbon and the measures relating to their mutual guardianship of subsidiarity, such as the Early Warning Mechanism. Such measures have increased the Commission's obligation to inform and give reasons to parliaments for their policies, while encouraging parliaments to develop the requisite scrutiny and control procedures. Most importantly, the role of national parliaments was explicitly acknowledged in Article 13 of the TSCG, which provided the basis for the creation of the Interparliamentary Conference on Economic and Financial Governance of the European Union. Although both these roles remain as yet rudimentary and untested, they are the subject of considerable policy interest at present (see, for example House of Lords [2014b]) and provide the beginnings of the sort of *demoi*cratic political constitution we have advocated for EMU.

## CONCLUSION

We have argued that the normative order of the EU requires that contracts between member states be seen as a two-level game, in which executives can only sign credible agreements as the duly authorized agents of their domestic peoples. We termed this *demoi*cratic structure ‘republican intergovernmentalism’. We argued that the attempt to view the neoliberal budgetary constraints of the Fiscal Compact as a supranational legal constitution not only conflicted with this normative order, but also was unjustifiable in denying the reasonable disagreements among both citizens and member states about economic policy. Instead, such measures have to be subject to a political constitution of a *demoi*cratic kind. The continuing role for national parliaments insisted on by the German Constitutional Court in its Lisbon Judgment and elsewhere (Federal Constitutional Court 2009) provide the basis for such a political constitutional framework for EMU.

## NOTE

1 Portuguese Constitutional Court Decisions 187/2013, 474/2013, 602/2013.
